# A Case of Pasteurella multocida Bacteremia in an Infant

**DOI:** 10.7759/cureus.93573

**Published:** 2025-09-30

**Authors:** Sarah Jong, Adriana Sarmiento Clemente, Charalene R Fisher, Linda Yarbrough, Jacob Filipek

**Affiliations:** 1 Medicine, University of Arkansas for Medical Sciences, Little Rock, USA; 2 Pediatrics/Infectious Disease, University of Arkansas for Medical Sciences, Little Rock, USA; 3 Pediatrics/Hospital Medicine, University of Arkansas for Medical Sciences, Little Rock, USA

**Keywords:** febrile infant, household pets, neonatal sepsis, pasteurella bacteremia, pasteurella multocida

## Abstract

We describe an uncommon case of a six-week-old male infant with *Pasteurella multocida *bacteremia. He presented to a tertiary care pediatric hospital emergency department (ED) in the American southeast (Arkansas, USA) with a new onset of fever and decreased oral (PO) intake. On exam, he was irritable, febrile, and tachycardic with no significant findings. Infectious workup was initially remarkable for an elevated procalcitonin of 7.25 ng/mL (reference range: 0.00-0.10 ng/mL) and a low white blood cell count of 3.2 K/cmm (reference range: 5.0-19.5 K/cmm). He was admitted and treated empirically with intravenous (IV) vancomycin, IV ceftriaxone, and IV acyclovir at meningitic dosing for possible sepsis and meningitis. A blood culture grew gram-negative rods, identified as *P. multocida*, while we were unable to interpret a bloody cerebrospinal fluid sample. Once beta-lactamase testing resulted negative, the patient completed a 14-day course of therapy with continuous IV penicillin per infectious disease recommendations. With antibiotics, pain management, and hydration, the patient improved clinically and was discharged home. Upon further questioning, parents reported that the patient was licked in the face by their pet cat but denied bites or scratches. In conclusion, *P. multocida* is an uncommon but reported cause of bacteremia and meningitis in infants. Exposure to dogs and cats is the leading risk factor; however, a bite wound or injury is not necessary for infection. This case demonstrates the high degree of clinical suspicion required to identify early presentations of *P. multocida *bacteremia and the importance for parents to be informed about the risk of infection and limit contact with pets during the first few months of life to prevent infection.

## Introduction

*Pasteurella multocida*, formerly known as *P. septica*, is the most common infecting organism in animal bites, affecting a wide variety of animal species [1]. It is a small, nonmotile gram-negative rod that can be grown on blood, chocolate, and Mueller-Hinton agars [1,2]. It is known to be part of the normal oral and respiratory flora of many animals, including dogs, cats, rabbits, rats, pigs, lions, tigers, and buffalo [1,2]. There are three subspecies of *Pasteurella*: *multocida*, *septica*, and *gallicida*, with *multocida* causing more than half of human *Pasteurella* infections, generally after bites, scratches, or licks from dogs or cats [1,2]. Bacteremia, which typically arises from hematogenous dissemination of the primary infection, poses a higher risk to immunodeficient individuals, such as neonates, and can lead to complications including sepsis and meningitis [1].

According to the current literature, most children diagnosed with *Pasteurella* meningitis are younger than one year of age and have had contact with a household pet [1]. They commonly present with fever, lethargy, and irritability, resembling clinical presentations seen with infections from other bacterial causes [1]. In the present study, we describe a case of a six-week-old male infant with *P. multocida* bacteremia and report a review of the literature, accounting for 67 other cases of *P. multocida* bacteremia, meningitis, or sepsis in infants since 1963.

This article was previously presented as a meeting abstract at the Southern Society for Pediatric Research 2025 Annual Meeting on February 13, 2025.

## Case presentation

A six-week-old male infant with a history of severe hypoxic ischemic encephalopathy, neonatal tremors, and gastroesophageal reflux disease presented to a tertiary care pediatric hospital emergency department (ED) in the American southeast (Arkansas, USA) from an outside hospital (OSH) with new-onset fever and decreased oral (PO) intake. The patient’s mother reported fever with a maximum temperature of 103°F measured at home, preceded by nasal congestion for two days and decreased PO intake, which prompted the visit to the OSH. The patient’s older brother was diagnosed with Streptococcal pharyngitis eight days prior.

Upon arrival at the OSH, the patient was irritable and in acute distress. Vital signs revealed a temperature of 102.7°F, heart rate of 150 beats per minute, and normal blood pressure, respiratory rate, and oxygen saturation for age. Infectious workup included a blood culture, urinalysis with culture, complete blood count and differential, procalcitonin and C-reactive protein levels, viral panel, Group A *Streptococcus* polymerase chain reaction test, and chest X-ray. The workup was initially remarkable for an elevated procalcitonin of 7.25 ng/mL (reference range: 0.00-0.10 ng/mL) and a low white blood cell count of 3.2 K/cmm (reference range: 5.0-19.5 K/cmm). The patient received a 20 mL/kg intravenous (IV) normal saline bolus, PO acetaminophen, and IV ceftriaxone before being transferred to our hospital.

Infectious workup was repeated in our ED, and cerebrospinal fluid (CSF) studies were obtained. The patient was admitted and treated empirically with IV vancomycin, IV ceftriaxone, and IV acyclovir at meningitic dosing for possible sepsis and meningitis. The patient underwent a traumatic lumbar puncture, showing inconclusive findings. Herpes simplex virus nucleic acid amplification testing of plasma and CSF was negative, so we discontinued acyclovir.

Blood culture at the OSH reported growth of gram-negative rods. We continued empirically treating with ceftriaxone and vancomycin pending identification of the pathogen. With antibiotics, pain management, and hydration, the patient began to improve clinically, remaining afebrile and increasing PO intake without emesis. The OSH identified the pathogen to be *P. multocida*. We discontinued vancomycin but continued to treat with ceftriaxone, pending antibiotic susceptibilities. Once beta-lactamase testing resulted negative, we discontinued ceftriaxone and completed a 14-day course of therapy with continuous IV penicillin per infectious disease recommendations. After demonstrating normal findings on magnetic resonance imaging of the brain, the patient was discharged home. During an in-depth history conducted by an infectious disease expert, the parents reported that the patient was licked in the face by their pet cat but denied bites or scratches.

## Discussion

*P. multocida* is an uncommon but reported cause of bacteremia and meningitis in the newborn period [1-3]. Exposure to dogs and cats is the leading risk factor for *P. multocida* bacteremia [1-3]; however, a bite wound or injury is not necessary for infection, as seen with our patient. Invasive infection occurs secondary to hematogenous dissemination from the primary infection site, which is often a soft tissue infection; however, it has also been suggested that fetal infection can occur secondary to maternal bloodstream infection and invasion through the placenta [4]. Dissemination can also occur secondary to oropharyngeal colonization after contact with secretions of a pet [4].

Studies have reported *Escherichia coli* as the most common pathogen causing bacteremia in young infants less than three months of age, with Group B *Streptococcus* and *Staphylococcus aureus* being the second and third most common [5,6]. Because of the lack of specific signs and symptoms that distinguish *P. multocida* infections from other causes of bacteremia and meningitis in this patient population, obtaining a detailed exposure history and performing a thorough workup, including cultures, is essential to making the diagnosis. Identification of these bacteria can be challenging because of their similar biochemical properties to other bacteria from the same family. Thus, for laboratories that rely on biochemical testing for identification, notification of the clinician’s suspicion can be helpful to differentiate [1].

**Figure 1:**
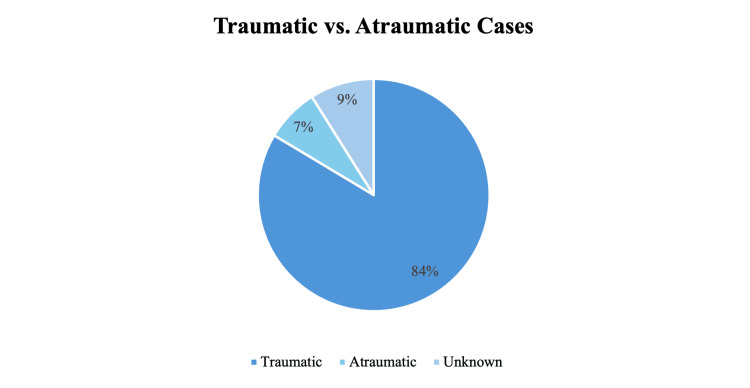
Distribution of traumatic and atraumatic cases of Pasteurella multocida bacteremia, sepsis, and meningitis reported in the literature. Image credit: Sarah Jong

In our review of the literature, we identified 67 reported cases of *Pasteurella* bacteremia, meningitis, and sepsis in infants since 1963 (Table 1) [7-67]. The baseline characteristics of each case were gathered, including the age and sex of each patient. The mean age was 3.16 months with a standard deviation of 3.32 months (ranging from birth to 12 months). Thirty-seven of the patients were males (55.22%), 24 were females (35.82%), and six were unknown (8.96%). We also documented whether direct trauma to the infant was reported and noted any exposure that may have predisposed the patient to infection. Surprisingly, 56 of the 67 cases (83.58%) did not involve direct trauma to the patient, with five being associated with trauma (7.46%) and six unknown (8.96%) (Figure 1). Exposure to animals was most common (73.13%), followed by vertical transmission (17.91%). Many of the cases determined to be caused by vertical transmission indicated maternal exposure to animals during pregnancy. Animals implicated included dogs, cats, rabbits, pigs, hens, roosters, and sheep.

**Table 1:** Demographics and characteristics of the 67 reported cases of invasive Pasteurella multocida infection. *F: female, M: male, U: unknown

Author	Year	Age	Sex	Trauma to infant?	Reported exposure
Whitmore and Whelan [7]	1963	6 months	F	No	Unknown
Bates et al. [8]	1965	64 hours	M	No	Vertical transmission
Controni and Jones [9]	1967	12 months	F	Unknown	Hen
Strand and Helfman [10]	1971	At birth	M	No	Vertical transmission
Repice and Neter [11]	1975	1 month	M	No	Cats
Slack and Walijee [12]	1975	5 months	M	No	Unknown
Chen et al. [13]	1976	7 weeks	F	No	Inconclusive
Bhave et al. [14]	1977	7 weeks	M	No	Dog saliva
Frutos et al. [15]	1978	25 days	U	No	Dog
Spencker et al. [16]	1979	7 weeks	M	Unknown	Cat/pig
Adenuga et al. [17]	1981	7 months	M	No	Unknown
Defawe et al. [18]	1981	2 days	M	No	Vertical transmission
Belardi et al. [19]	1982	10 months	M	Yes	Dog bite
Prince-David et al. [20]	1982	11 days	U	Unknown	Unknown
Thompson et al. [21]	1984	3 weeks	M	No	Cat
Weber et al. [22]	1984	7 weeks	M	No	Cats
Steinbok et al. [23]	1985	2 weeks	F	Yes	Dog bite
Clapp et al. [24]	1986	5 months	F	No	Dog saliva
Clapp et al. [24]	1986	5 weeks	F	No	Dog saliva
King and Sills [25]	1987	2 days	U	No	Vertical transmission
Tessin et al. [26]	1987	11 months	F	Yes	Cat scratch
Levy et al. [27]	1989	5 weeks	F	Unknown	Unknown
Costa-Cruz et al. [28]	1990	8 weeks	M	No	Dog saliva
Wong et al. [29]	1992	At birth	M	No	Vertical transmission
Wong et al. [29]	1992	At birth	F	No	Vertical transmission
Giacomini et al. [30]	1993	1 month	M	No	Dog, rabbit
Hillery et al. [31]	1993	2 days	M	No	Vertical transmission
Pilorget et al. [32]	1994	27 days	U	Unknown	Dog and cat
Boocock and Bowley [33]	1995	5 weeks	M	No	Dog and cat
Miller and Gray [34]	1995	17 days	M	No	Cat
Blackwood et al. [35]	1996	3.5 months	M	No	Dogs and kittens
Challapalli and Covert [36]	1997	2 days	U	No	Vertical transmission
Escande et al. [37]	1997	At birth	M	No	Vertical transmission
Schuur et al. [38]	1997	4 months	F	Yes	Cat scratches
Kouppari et al. [39]	1999	29 days	F	No	Cat scratch
Wade et al. [40]	1999	7 weeks	M	No	Dog saliva
Zaramella et al. [41]	1999	At birth	M	No	Vertical transmission
Boerlin et al. [42]	2000	1 month	U	No	Dogs and cat
Mestre et al. [43]	2001	10 days	F	Unknown	Dog
Perrin et al. [44]	2003	3 months	M	No	Dog saliva
Ahmed and Steele [45]	2004	6 weeks	M	No	Dog saliva
Hirsh et al. [46]	2004	20 days	M	No	Cats
Best et al. [47]	2005	19 days	F	No	Cats
Koranyi et al. [48]	2005	9 weeks	M	No	Cat saliva
Cohen-Adam et al. [49]	2006	4 weeks	F	No	Dog and cat
Goytia et al. [50]	2006	26 days	M	No	Cat
Haase et al. [51]	2006	3 weeks	M	No	Cat
Puwanant and Chanvitan [52]	2006	2 days	M	No	Vertical transmission
Guillet et al. [53]	2007	2 months	M	No	Sheep
Guillet et al. [53]	2007	2 months	M	No	Sheep
Pace and Attard-Montalto [54]	2008	At birth	F	No	Vertical transmission
Haybaeck et al. [55]	2009	2 months	M	No	Dog
Kobayaa et al. [56]	2009	25 days	M	No	Cat saliva
Kobayaa et al. [56]	2009	30 days	F	No	Dog saliva
Spadafora et al. [57]	2011	25 days	M	No	Rooster
Siahanidou et al. [58]	2012	17 days	F	No	Dog
Guet-Revillet et al. [59]	2013	5 weeks	F	No	Dog
Guet-Revillet et al. [59]	2013	6 weeks	F	No	Dog
Guet-Revillet et al. [59]	2013	8 weeks	F	No	Cat
Guet-Revillet et al. [59]	2013	11 weeks	M	No	Cat
Wood et al. [60]	2013	25 days	M	No	Cat
Aguado et al. [61]	2014	22 days	F	No	Dog
Yamaguchi et al. [62]	2014	23 days	F	No	Cat
Boyanton et al. [63]	2016	17 days	M	No	Dog and cats
Ryan and Feder [64]	2019	12 days	M	No	Dog saliva
Jha and Kalyoussef [65]	2021	7 weeks	F	No	Dogs and cats
Slehria et al. [66]	2022	33 days	F	No	Dog saliva

## Conclusions

This case report and review emphasize the high degree of clinical suspicion required to identify early presentations of *P. multocida* invasive infection, as the vast majority of published cases of *P. multocida* in infants did not involve direct trauma to the patient. Eliciting a detailed, in-depth history is crucial when developing a differential diagnosis for febrile infants. Finally, parents of young infants should be informed about the risks and potential associations between household pets and infections in their infants, with primary care providers playing a key role in educating families on risk of exposure and strategies to minimize it.
